# Standard multiple imputation of survey data didn’t perform better than simple substitution in enhancing an administrative dataset: the example of self-rated health in England

**DOI:** 10.1186/s12982-021-00099-z

**Published:** 2021-07-24

**Authors:** Frank Popham, Elise Whitley, Oarabile Molaodi, Linsay Gray

**Affiliations:** MRC/CSO Social and Public Health Sciences Unit, University of Glasgow, 200 Renfield Street, Glasgow, G2 3AX UK

**Keywords:** Surveys, Census, Validation, Multiple imputation

## Abstract

**Background:**

Health surveys provide a rich array of information but on relatively small numbers of individuals and evidence suggests that they are becoming less representative as response levels fall. Routinely collected administrative data offer more extensive population coverage but typically comprise fewer health topics. We explore whether data combination and multiple imputation of health variables from survey data is a simple and robust way of generating these variables in the general population.

**Methods:**

We use the UK Integrated Household Survey and the English 2011 population census both of which included self-rated general health. Setting aside the census self-rated health data we multiply imputed self-rated health responses for the census using the survey data and compared these with the actual census results in 576 unique groups defined by age, sex, housing tenure and geographic region.

**Results:**

Compared with original census data across the groups, multiply imputed proportions of bad or very bad self-rated health were not a markedly better fit than those simply derived from the survey proportions.

**Conclusion:**

While multiple imputation may have the potential to augment population data with information from surveys, further testing and refinement is required.

**Supplementary Information:**

The online version contains supplementary material available at 10.1186/s12982-021-00099-z.

## Background

Many countries rely on surveys to estimate the prevalence and incidence of health measures and health-related exposures that are not routinely collected for the whole population. These estimates are important for including resource planning and monitoring the effectiveness of national strategies. However survey samples are rarely representative of the target population. In particular, individuals who are older, sick, disadvantaged, or display poor health behaviours are often under-represented in surveys [1], an important omission as these are often the groups of most interest when identifying priorities for health and economic policies. In addition, many surveys are too small to allow sub-population analyses. The lack of alignment between survey and population data is growing as a result of declining response levels over time [2]. Common solutions to this problem include using inverse probability weighting and/or post stratification weights to correct survey-based results for any departure from representativeness of the target population arising from the sampling design and/or response level. However, this approach is often limited as weights are usually based on a restricted combination of population characteristics (typically age and sex). More elaborate bespoke techniques have been used to some effect to obtain superior population and sub-population level estimates from surveys [3–5], for example using multilevel models to estimate variables of interest in terms of respondents’ characteristics and also those of the area in which respondents live, with results from these models then weighted by the frequency of the modelled characteristics in the target population [4, 6]. While this type of approach offers an improvement on traditional weighting, including more variables common to the survey and target population, their use is still limited, in particular producing group level estimates rather than the individual data required for further statistical analysis.

In contrast to specific surveys, administrative data, for example population censuses, are often highly representative of the target population. However, in order to maintain high response levels, reduce participant burden, and keep costs down, a limited number of questions are asked [7]. For example, many population censuses do not include smoking in spite of its recognised importance in determining health outcomes [8]. Estimates of the effect of smoking and the impact of interventions or policies aimed at its reduction are therefore based on survey data, with accompanying problems of smaller samples and non-response, meaning that results may be unrepresentative of target populations and that time trends and estimates for certain subgroups and small geographic areas, often those of most interest in terms of improving health and reducing inequalities, are imprecise [9]. Indeed, government departments and others have advocated for smoking to be included in the UK census, but this has not been adopted, and is unlikely to be included in the future [10]. There is therefore a need for a simple, transparent and robust approach that allows analysis of representative data, such as population censuses, to be carried out as if smoking, or any other variable, was included.

One such method is to impute the variable of interest into the census using data from a survey in which the variable is included and applying standard multiple imputation (MI) techniques [11]. This idea is not new [12] but we are not aware of any instances of its practical application. We have therefore tested the validity of the approach using data from the English population census and a corresponding English survey, using the example of general self-rated health as this is available in both datasets, allowing direct comparison of data imputed into the census from the survey with the actual census data. These census data are widely used by government, policy-makers and researchers to explore socio-economic and geographical patterning of health [13] and their possible enhancement from survey data has great potential to improve health, economic and social policy.

## Methods

### Datasets

The census dataset is the Census Microdata Individual Safeguarded Sample (Regional): England and Wales [14], a 5% stratified sample of the April 2011 census that is proportionally representative of the population of those countries. This dataset provides anonymised individual level data on a wider range of variables than standard census tables, based on the whole population, which are typically limited to cross-tabulations of three or four variables. Self-rated health in the census was based on the question “How is your health in general? (Very good, Good, Fair, Bad, Very bad)”. Survey data are from the Integrated Household Survey [15], which combines core questions asked in the General Lifestyle Survey, the Living Cost and Food Survey, and the Labour Force/Annual Population Survey. We chose the April 2011 to March 2012 dataset as this was contemporaneous with census data and question wording was similar to that in the 2011 census. Self-rated health in the Integrated Household Survey was based on the question “How is your health in general; would you say it was … (Very good, Good, Fair, Bad, or Very bad)?”. Both the survey and census data were obtained from the UK data archive.

The population frame for both datasets was:

- Those living in England, as each country’s census varied slightly and there are national differences in how self-rated health is conceptualised in relation to objective health [16]; the choice of the largest UK country therefore simplified the analysis.
- Those aged 25–64, as highest qualification was included in our MI and fewer older people had formal qualifications.
- Those living in non-communal households and classed as usual residents (so excluding students usually living away from home and visitors), as the surveys do not comprehensively cover communal establishments.

Variables forming the basis of the MI were chosen to be predictors of self-rated health that were available and able to be harmonised across the two datasets (Table 1). Key variables (those included in the MI and also forming the basis of stratification in the examination of health patterning) were age, sex, housing tenure, and English region. Auxiliary variables (those included in the MI but not in the subsequent stratification) were education, marital status, country of birth, and ethnicity. The ethnicity variable presented in Table 1 is a five-category summary for convenience; the variable used in the imputation model split respondents into 18 distinct groups. A small number of survey records that were missing any harmonised variables were dropped to maintain the simplicity of the subsequent imputation; there were no missing variables for the census records as this is subject to imputation by the census offices before release. Before imputation the harmonised datasets were combined into one file and the self-rated health variable deleted from the census records, leaving a dataset with “missing” self-rated health to be imputed from the remaining (survey) data.

**Table 1:** Distribution of harmonised key and auxiliary imputation variables in census and survey datasets

	Census data (n (%)) N = 1,390,094	Survey data (n (%)) N = 134,083
Key variables		
Sex		
Male	687,812 (49.5)	64,166 (47.9)
Female	702,282 (50.5)	69,817 (52.1)
Age group		
25–29	179,770 (12.9)	14,742 (11.0)
30–34	173,146 (12.5)	15,674 (11.7)
35–39	176,241 (12.7)	16,107 (12.0)
40–44	193,181 (13.9)	18,178 (13.6)
45–49	192,828 (13.9)	18,596 (13.9)
50–54	169,063 (12.2)	17,300 (12.9)
55–59	148,525 (10.7)	15,998 (11.9)
60–64	157,340 (11.3)	17,488 (13.0)
Housing tenure		
Owns home outright	297,630 (21.4)	30,938 (23.1)
Mortgage/shared ownership	637,581 (45.9)	62,250 (46.4)
Private rent	256,592 (18.5)	21,518 (16.1)
Social rent	198,291 (14.3)	19,377 (14.5)
English region		
North east	67,752 (4.9)	10,994 (8.2)
North west	183,198 (13.2)	21,460 (16.0)
Yorkshire and the Humber	136,179 (9.8)	14,438 (10.8)
East Midlands	117,676 (8.5)	9,064 (6.8)
West Midlands	143,120 (10.3)	14,076 (10.5)
East of England	152,850 (11.0)	11,856 (8.8)
London	230,677 (15.6)	17,830 (13.3)
South east	223,883 (16.1)	20,509 (15.3)
South west	134,759 (9.7)	13,856 (10.3)
Auxiliary variables		
Highest educational qualification^a^		
Higher education	467,010 (33.6)	43,244 (32.3)
School (advanced)	165,525 (11.9)	26,421 (19.7)
School (standard)	413,096 (29.7)	36,913 (27.5)
Other qualifications	128,137 (9.2)	10,417 (7.8)
No formal qualifications	216,326 (15.6)	17,088 (12.7)
Marital status		
Single	410,131 (29.5)	35,650 (26.6)
Married	747,394 (53.8)	77,440 (57.8)
Civil partnership	4327 (0.3)	486 ( 0.4)
Divorced	153,731 (11.1)	13,559 (10.1)
Widowed	24,058 (1.7)	2412 (1.8)
Separated	50,453 (3.6)	4536 (3.4)
Country of birth		
UK	1,141,684 (82.1)	112,135 (83.6)
European Union	74,891 (5.4)	6434 (4.8)
Other	173,519 (12.5)	15,514 (11.6)
Ethnicity		
White	1,196,262 (86.1)	117,832 (87.9)
Mixed race	19,874 (1.4)	973 (0.7)
Asian	110,598 (8.0)	9729 (7.3)
Black	47,930 (3.5)	3757 (2.8)
Other	15,430 (1.1)	1792 (1.3)

^a^Higher education: Degree level qualification (or equivalent)/Higher education qualification below degree level; School (advanced): A Level/Higher/Advanced Diploma/Progression Diploma/ONC/National Level BTEC; School (standard): O Level or GCSE equivalent/O Grade or CSE equivalent/Standard Grade/Higher or Foundation diploma; Other qualifications: including foreign qualifications below degree level

### Multiple imputation

MI is a flexible, simulation-based statistical technique for handling missing data that allows fully for the uncertainty arising from the missingness [17]. It is appealing compared with other methods as it makes estimation of variances and confidence intervals relatively straightforward. MI generates multiple sets of plausible values under the missing at random assumption [18]. Under this assumption, we consider that the missing self-rated health values from the census data are sufficiently informed by the key and auxiliary variables from both the census and survey data along with the self-rated health observations for the survey participants. In this case the missing self-rated health data in the census were imputed from MI models that included the main effects of all variables and all possible interactions between the key variables (age, sex, housing tenure, and English region). Four different models were explored: (a) standard logistic regression comparing very bad/bad versus fair/good/very good self-reported health; (b) poisson regression also based on dichotomous self-rated health, giving estimates of risk rather than odds of very bad/bad self-reported health; (c) ordinal logistic regression of the five ordered categories of self-rated health under the proportional odds assumption, considering the distance between each category to be equivalent; and (d) multinomial logistic regression of the five category self-rated health variable with no assumptions regarding the spacing or order of categories. Census records in the pooled dataset accounted for about 90% of the observations, therefore 90 imputations were carried out in line with recommendations that the number of imputations reflects the percentage missing [19]. The imputed datasets were analysed using standard techniques with resulting estimates and standard errors averaged across all 90 according to the “Rubin rules” [11], details of which are presented in Additional file 1. Imputations (and analyses) were performed in Stata v14 using the mi impute command [20].

### Assessment

A priori (see initial analysis plan [21]) the performance of the imputation approach was assessed by comparing proportions of bad or very bad self-rated health from imputed versus actual census data across all 576 combinations of the key variables (8 age groups × 2 sex categories × 4 housing tenure categories × 9 English regions) as similar three and four way tables are used to realise census results. For comparison, these were compared with missing census proportions derived directly from the survey (without imputation). Associations between the actual census proportions of bad health and those from MI models (or directly from the survey) were explored graphically and using lines of best fit to these data (weighted according to the size of each age-sex-tenure-region category). Specifically, we considered the extent to which the lines of best fit deviated from the line of equality (y = x) with slope = 1 and intercept = 0, representing (theoretical) perfect agreement. We also considered the strength of the linear association between actual and imputed proportions of bad health using correlation coefficients.

## Results

The original census and survey datasets comprised of 2,848,155 and 374,218 records respectively, of which 1,390,094 (49%) and 134,717 (36%) were respondents aged 25–64, living in England and who were usual residents in non-communal households. In total 634 (0.5%) respondents in the survey dataset had missing values for at least one key or auxiliary variable and they were omitted from analyses, leaving a total of 134,083 survey respondents in the analytical dataset. Distributions of imputation variables in the census and survey are presented in Table 1. Distributions were broadly similar in the two datasets with survey respondents slightly older, more educated, and more likely to be female, own their home, and be married than those from the census.

**Fig. 1:**
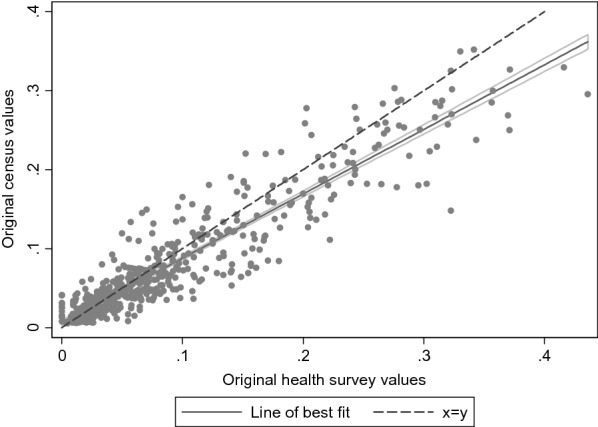
Comparison of proportion of bad or very bad self-rated health in original census data versus survey data

Census and survey responses to questions on self-rated health are presented in Table 2. Overall, survey respondents were less positive about their health, with 78% rating it as good or very good compared with 83% of the census. Figure 1 presents a scatter plot of the proportion of survey versus census respondents in each of the 576 combinations of age, sex, tenure and region who rated their health as bad or very bad. The dashed line is the line of equality, representing perfect agreement between census and survey measures, while the solid line (shaded line) shows the regression line of best fit (95% confidence interval) describing the association between the two. The intercept and slope of this line of best fit are presented in Table 3. There was a strong linear relationship between the proportions in the two datasets (correlation = 0.93; Table 3). However, the survey overestimated the proportion of respondents with bad or very bad self-rated health, as evidenced by the lack of correspondence between the regression line (intercept (95% confidence interval): 0.01 (0.00, 0.01); slope (95% confidence interval): 0.82 (0.79, 0.84)) and the line of equality.

**Table 2:** Overall distribution of self-rated health in original census data versus data from or imputed from survey data

	Census data	Raw survey data	Imputed from survey data			
			Standard logistic regression	Poisson regression	Ordinal logistic regression	Multinomial logistic regression
	% (N)	% (N)	% (95% CI)	% (95% CI)	% (95% CI)	% (95% CI)
Self-rated health						
Very good	42.6 (592,633)	37.9 (50,792)	–	–	38.0 (37.7, 38.3)	38.2 (37.9, 38.5)
Good	40.2 (558,445)	40.5 (54,281)			40.2 (39.8, 40.5)	40.1 (39.8, 40.4)
Fair	12.1 (167,813)	15.4 (20,707)			15.6 (15.4, 15.8)	15.2 (14.9, 15.4)
Bad	4.0 (55,535)	4.8 (6440)			4.8 (4.7, 4.9)	4.9 (4.8, 5.0)
Very bad	1.1 (15,668)	1.4 (1863)			1.4 (1.3, 1.4)	1.6 (1.5, 1.8)
Self-rated health						
Very good/good/fair	94.9 (1,318,891)	93.8 (125,780)	93.7 (93.6, 93.9)	93.8 (93.7, 93.9)	93.8 (93.7, 94.0)	93.5 (93.3, 93.7)
Bad/very bad	5.1 (71,203)	6.2 (8303)	6.3(6.1, 6.4)	6.2 (6.0, 6.3)	6.2 (6.0, 6.3)	6.5 (6.3, 6.7)

**Table 3:** Linear associations between proportion of bad or very bad self-rated health across 576 groups comparing original census data with data from or imputed from survey data

	Raw survey data	Imputed from survey data			
		Standard logistic regression	Poisson regression	Ordinal logistic regression	Multinomial logistic regression
Linear regression line					
Intercept (95% CI)	0.01 (0.00, 0.01)	0.00 (− 0.00, 0.00)	0.00 (− 0.00, 0.00)	− 0.01 (− 0.01, − 0.00)	− 0.00 (− 0.01, − 0.00)
Slope (95% CI)	0.82 (0.79, 0.84)	0.82 (0.79, 0.84)	0.80 (0.78, 0.82)	1.00 (0.98, 1.03)	0.83 (0.81, 0.85)
Correlation	0.93	0.95	0.95	0.96	0.95

**Fig. 2:**
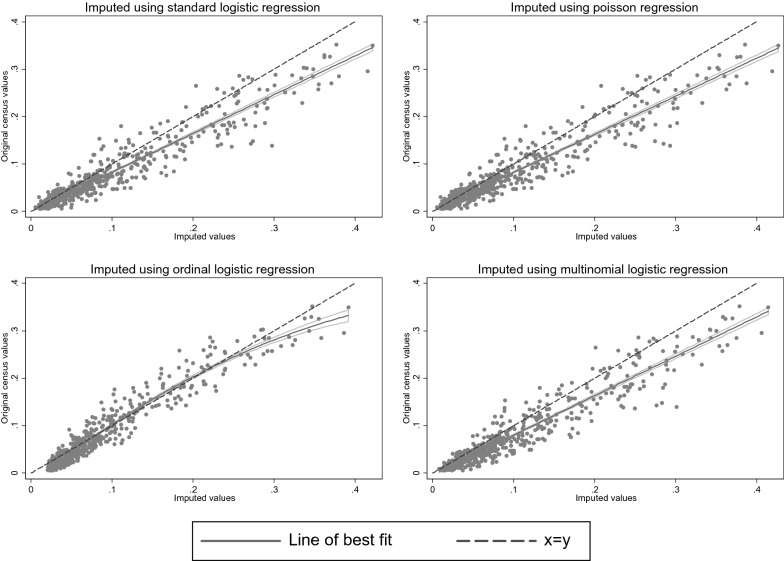
Comparison of proportion of bad or very bad self-rated health in original census data versus data imputed from survey data

Similar results for the multiply imputed data are presented in Fig. 2 (Tables 2 and 3). The overall distribution of bad or very bad self-rated health imputed into the census from survey data using standard logistic or poisson regression was very similar to that for the raw survey data (6.3% and 6.2% of imputed census data versus 6.2% of raw survey data were bad or very bad) and, therefore, differed from the original census values (5.1%). Results for the 576 combinations of age, sex, tenure and region (Fig. 2, top left and right) were also very similar to those for raw survey data, with a strong linear relationship, but were generally overestimations relative to the original census values (logistic intercept: 0.00 (− 0.00, 0.00); slope: 0.82 (0.79, 0.84); correlation: 0.95; poisson: 0.00 (− 0.00, 0.00); 0.80 (0.78, 0.82); 0.95). The overall distribution of self-rated health imputed into the census using ordinal logistic regression was, again more similar to the original survey data than the original census data (6.2% bad or very bad self-rated health). Initially, it seemed that the association for the 576 categories was a better fit to the original census data (intercept: − 0.01 (− 0.01, − 0.00); slope: 1.00 (0.98, 1.03)) than that from the raw survey data. However, while there was reasonable linear agreement between values in the middle of the range, the imputed data substantially overestimated the proportion of bad or very bad self-rated health at the lower and upper ends of the distribution and, in practice, a quadratic model was a better fit in describing the association between imputed and original census values (Fig. 2, bottom left). Results for data imputed into the census using multinomial logistic regression were again very similar to those for the raw survey data (6.5% bad or very bad self-rated health; intercept: − 0.00 (− 0.01, − 0.00); slope: 0.83 (0.81, 0.85); correlation: 0.95; Fig. 2, bottom right).

## Discussion

Our aim was to assess whether applying MI to data from the Integrated Household Survey would provide a simple, accessible and robust means of predicting the (known) prevalence of bad or very bad self-rated health in the UK census. The major strength of our analysis is the ability to test the performance of MI against a known result; often such cross-validation is missing from assessments of methods to estimate population parameters. Our results suggest that distributions of imputed self-rated health were more similar to the original survey than the census data and there was little additional benefit offered by standard MI. This highlights the importance of comparison with known data in the development of tools for enhancing routine datasets.

Although we included a wide range of predictors of self-reported health and all possible interactions among our four key variables, the imputation process was not sufficient to completely account for differences between the survey and census populations. As multiple imputation tends to perform less well with higher levels of missing observations, [22] the large percentage (90%) of records in the pooled dataset that were census ones may partially explain the marginal benefit of our application. Including additional interactions with auxiliary variables might have improved the model but this was not possible in our standard software (Stata v14) and may require more specialist software, making it unsuitable for general application. The accuracy of the current model might be improved by incorporating machine learning into the process, enabling the identification of an optimal prediction model with a rich array of higher order terms beyond selected two-way interactions. Additionally, the adoption of a generalized linear mixed model based approach allowing for cluster specific weighting may also enhance accuracy. Likewise, the incorporation of survey design weights in multiple imputation models could be used to improve survey-based estimates [23, 24]. However, these more complex approaches would limit accessibility. However, perhaps the most important limitation is that the method relies on having harmonised variables common to both datasets. In the current analysis, variables were chosen for their known associations with self-rated health and on the basis that they could be harmonised across the census and the survey and it is possible that important variables were omitted from the current models. The strength of administrative data such as censuses lies in their representativeness but this is often tempered by the need to restrict the number of questions asked. The range of variables available for MI will therefore be limited by availability and comparability across datasets and this may restrict the practical applications of the approach in some circumstances.

There is concern that survey-based estimates of population parameters are not sufficiently robust to inform resource planning and policy development and assessment, especially as sub-populations who are most sick, most disadvantaged, and with the least healthy lifestyles are increasingly underrepresented [2]. In addition to limiting the generalisability of the findings from analyses of survey data, the groups that are most often missing are those of greatest potential importance in determining economic and public health policy. Many surveys derive and provide general weights in order to make results from (weighted) analyses representative of the population from which the data are drawn. However, these weights are frequently based on just a few population characteristics (often simply age and sex) and may be limited in their capacity to adequately correct estimates [25, 26]. Increased access to administrative databases and other sources of “big data”, with their vastly more extensive population coverage, creates a potential opportunity to overcome these shortcomings [27, 28]. However, these datasets often do not include the range or quality of variables that surveys do, for example the UK census does not include questions on smoking, generating a need for alternative analytical methods to bridge the gap.

## Conclusion

We have explored an easily applied and accessible method using MI models to impute individual level data, amenable for use in further modelling, from a survey into a larger but less comprehensive administrative dataset. However, our results demonstrate that the practical application of this approach is not straightforward. We do not discount its use in the context of enhancing routine datasets. However, further work is clearly needed to explore its validity and application in this context and, in particular, it is important to understand how to identify and develop the best imputation models and how to select the most useful surveys and variables for inclusion in them. We recommend following our example of comparing imputed survey values with those already known in the administrative data in order to more rigorously assess the performances and validity of different approaches and datasets.

## Supplementary Information

**Additional file 1: Appendix. **

## Data Availability

The datasets used and/or analysed during the current study are available from the corresponding author on reasonable request.
