# Supplementary material for: Standard multiple imputation of survey data didn’t perform better than simple substitution in enhancing an administrative dataset: the example of self-rated health in England
Source: Emerg Themes Epidemiol. 2021 Jul 24;18:9. doi: 10.1186/s12982-021-00099-z (PMC8310590; doi:10.1186/s12982-021-00099-z)
Supplement: Supplementary file 1 — Additional file 1: Appendix. [file 12982_2021_99_MOESM1_ESM.docx]

Appendix

Let $Z$ denote the rectangular data set with $Z_{k}$ the partially observed variable of interest, the last of which, $Z_{k}$, is fully observed in the survey data and (following removal) entirely missing in the census data. Then, let $Z=Z^{S}\bigcup Z^{C}$ where $Z^{S}= (z_{S_{i}j})$ denotes a $(n_{S} x K)$ rectangular sub data set, with $S_{i}$th row $z_{S_{i}}=(z_{S_{i}1},\ldots, z_{S_{i}K})$ where $z_{S_{i}j}$ is the value of variable $Z_{j}$ for individual $S_{i}$ in a sample of $n_{S}$ subjects in the survey. Equivalently, let $Z^{C}=(z_{C_{i}j})$ denote a $(n_{C} x K)$ rectangular sub data set, with $C_{i}$th row $z_{C_{i}}=(z_{C_{i}1},\ldots, z_{C_{i}K})$ where $z_{C_{i}j}$ is the value of variable $Z_{j}$ for individual $C_{i}$ in a sample of $n_{C}$ subjects in the census. Data for all variables are thus defined, for example: $Z_{k}={(z_{S_{1}K},\ldots, z_{S_{n_{s}}K},z_{C_{1}K},\ldots, z_{C_{n_{c}}K})}^{T}$.

Let $R=R^{S}\bigcup R^{C}$, where $R^{S}=(r_{S_{i}j})$ and $R^{C}=(r_{C_{i}j})$, define the missing-data indicator matrix such that $r_{C_{i}j}=0$if $z_{C_{i}j}$ is missing and $r_{C_{i}j}=1$if $z_{C_{i}j}$ is present; here, $r_{C_{i}K}=0, {\forall z}_{C_{i}K}$since the $z_{C_{i}K}$ are missing for every $C_{i}$ and $r_{C_{i}j}=1, {\forall z}_{C_{i}j}$for $j$=1,…K-1 and ${r_{S_{i}j}=1 \forall z}_{S_{i}j}$. Let $Z_{\mathrm{obs}}$ denote the observed components and $Z_{\mathrm{mis}}$ the missing components of $Z$. The missing data mechanism is then characterised by the conditional distribution of *R* given $Z$, say $p\left( R | Z,ø \right)$, where *ø* denotes unknown parameters.

Once all missing values have been imputed, each imputed data set can be analysed using standard techniques and the results combined using standard rules.(11) Using Rubin’s framework, let $\theta$ denote the scalar quantity to be estimated, in this case the proportion of very bad/bad self-reported health. Let $\hat{\theta}$ = $\hat{\theta}\left( Z_{obs},Z_{mis} \right)$ denote the statistic that would be used to estimate $\theta$ if complete data were available and let $W$ = $W\left( Z_{obs},Z_{mis} \right)$ be its squared standard error. We assume that, with complete data, tests and intervals based on the normal approximation $\frac{(\hat{\theta}-\theta)}{\sqrt{W}}\sim N(0,1)$are appropriate. Suppose that we generate $M$ repeated independent imputations $Z_{mis}^{(1)},\ldots,Z_{mis}^{(M)}$ under one model. From these we calculate the imputed-data estimates $\hat{\theta}^{(m)}$ = $\hat{\theta}\left( Z_{obs},{Z_{mis}}^{(m)} \right)$ along with their estimated variances $W^{(m)}$ = $W\left( Z_{obs},{Z_{mis}}^{(m)} \right)$, where $m=1,\ldots,M$. The overall estimate of $\theta$ is simply the average $\bar{\theta}=m^{-1}\sum\hat{\theta}^{\left( m \right)}.$
